# Using vibrational infrared biomolecular spectroscopy to detect heat-induced changes of molecular structure in relation to nutrient availability of prairie whole oat grains on a molecular basis

**DOI:** 10.1186/s40104-016-0111-y

**Published:** 2016-09-09

**Authors:** M. D. Mostafizar Rahman, Katerina Theodoridou, Peiqiang Yu

**Affiliations:** 1 Ministry of Agriculture Strategic Research Chair in Feed R&D, Department of Animal and Poultry Science, College of Agriculture and Bioresources, The University of Saskatchewan, 51 Campus Drive, Saskatoon, SK S7N 5A8 Canada; 2 The United Graduate School of Agricultural Sciences, Iwate University, Morioka, Iwate 020-8550 Japan; 3 Institute for Global Food Security, Queen’s University Belfast, Clooreen Park, Malone Road, BT95HN Belfast, UK

**Keywords:** Dry roasting, Feed processing, Microwave irradiation, Modeled cereal grains, Molecular structure, Nutrient availability

## Abstract

**Background:**

To our knowledge, there is little study on the interaction between nutrient availability and molecular structure changes induced by different processing methods in dairy cattle. The objective of this study was to investigate the effect of heat processing methods on interaction between nutrient availability and molecular structure in terms of functional groups that are related to protein and starch inherent structure of oat grains with two continued years and three replication of each year.

**Method:**

The oat grains were kept as raw (control) or heated in an air-draft oven (dry roasting: DO) at 120 °C for 60 min and under microwave irradiation (MIO) for 6 min. The molecular structure features were revealed by vibrational infrared molecular spectroscopy.

**Results:**

The results showed that rumen degradability of dry matter, protein and starch was significantly lower (*P* <0.05) for MIO compared to control and DO treatments. A higher protein α-helix to β-sheet and a lower amide I to starch area ratio were observed for MIO compared to DO and/or raw treatment. A negative correlation (−0.99, *P* < 0.01) was observed between α-helix or amide I to starch area ratio and dry matter. A positive correlation (0.99, *P* < 0.01) was found between protein β-sheet and crude protein.

**Conclusion:**

The results reveal that oat grains are more sensitive to microwave irradiation than dry heating in terms of protein and starch molecular profile and nutrient availability in ruminants.

## Background

Nowadays, there is high demand for the development of highly productive animals and as a result the energy and protein availability from the feed need to be increased. To meet this request, cereal grains (i.e., oat, barley) are generally fed to ruminant in order to increase the nutrient density of diet. On the other hand, a problem remains, the rapidly and extensively degradation of their starch and protein in the rumen. This may cause serious digestive disorders and nutritional imbalance, which may also result in nitrogen loss and inefficient use of dietary energy [1–5]. To improve the utilization of cereal grains and the availability of their nutrients, heat treatments have been used [1, 6–10]. Although heat treatments are commonly used during the processing of cereal grains, just few research studies exist on how heat treatments would affect their nutrient molecular structures and utilization interaction by ruminants. The investigation of molecular structure that are related to protein and starch is often crucial to understand their digestive behaviour, nutritive quality, and availability in ruminants [6, 7, 11, 12]. Oat grains (*Avena sativa* L.) are a good source of protein, unsaturated fatty acids, and phenolic compounds as well as dietary fibre and starch. Although oats have a slightly lower energy range than most of the other grains, they have high fibre content, and are considered a safer grain to feed ruminants than either wheat or barley [13, 14].

Fourier-transformed infrared-vibration spectroscopy is capable to detect structure features on a molecular basis, including protein, carbohydrate, lipid related functional groups [1]. However, to our knowledge, there is few study on the interaction between nutrient availability and molecular structure changes induced by different processing methods in dairy cattle [15–19]. The objective of this study was to investigate whether molecular structures that are related to protein and starch are highly affected by different heat processing methods using attenuated total reflectance-Fourier-transformed infrared-vibration spectroscopy (ATR-FT/IR) [20], and to study the heat-induced changes in nutrient profile and availability in relation to molecular structure changes in ruminants. The results of this study will allow us to select the most suitable heat processing method for oat cereal grains, in order to increase their efficiency for ruminants.

## Methods

Animal trails were approved by the University of Saskatchewan Animal Research Ethics Board (AREB) with ANIMAL USE PROTOCOL # 19910012 and were conducted in accordance with the Canadian Council of Animal Care guidelines in 1993 [21].

### Oat seed processing method

Oat grains (*Avena sativa* L. cv. ‘CDC Dancer), harvested in two different years were used for feed protein study and obtained from the Crop Development Centre, University of Saskatchewan, Canada. For heating processing 1 kg of oat grains was roller milled through 0.20 mm dies, divided into 3 portions and allowed for 3 different treatments. Control sample (RO) was kept raw. Dry heated sample (DO) was heated in an air-draft oven at 120 °C for 60 min. Microwave irradiated sample (MIO) was heated in a microwave oven for 6 min in which sample was taken out intermittently and mixed in every 2 min.

### ATR-FT/IR molecular spectroscopy

Each oat seed sample were finely ground using a fit with a 0.5 mm screen. The molecular spectral data of oat seed were collected and corrected with the background spectrum using Jasco ATR-FT/IR 4200 (Jasco Inc., Easton, MD Corp., Tokyo, Japan). The spectra were generated with mid-IR (ca. 4,000–800 cm^−1^) and fingerprint region (ca. 1,800–800 cm^−1^) with spectral resolution of 4 cm^−1^. The ATR-FT/IR spectral data were analysed using OMNIC 7.3 (Spectra-Tech Inc., Madison, WI) software. Chemical functional groups were identified according to published results [11]. The regions of specific interest in this present study included the protein amide I, II, and protein structure of α-helix and β-sheet and starch in the IR regions of approximately 1,715–1,480 cm^−1^ and 949–1,062 cm^−1^, respectively. The ratios of amide I and II, α-helix and β-sheet and amide I and starch spectral intensities were calculated.

### Rumen degradation

For the rumen degradation study, the standard Dept in situ method was applied with dry Holstein cows with flexible rumen cannula (10 cm i.d.; Bar Diamond Inc., Parma, ID, USA). The animals were housed in the research barn at the University of Saskatchewan. The cows were given ad libitum access to water and 15 kg (as fed) of a totally mixed ration (TMR) twice daily (7.5 kg/feeding) at 0800 and 1600 h, formulated to meet or exceed NRC requirements [22]. Seven grams of an individual ground sample were weighed into each pre-weighed and numbered nylon bag (10 cm × 20 cm; Nitex 03-41/31 monofilament open mesh fabric, Screentec Corp., Mississauga, ON) with the pore size of 40 μm. These bags were tied about 2 cm below the top, allowing a ratio of sample size to bag surface area of 19 mg/cm^2^. Samples were incubated in the rumen for 16 h in dry Holstein cows with two runs. After the incubation, the bags were removed from the rumen and rinsed under a cold stream of tap water without detergent to stop microbial enzymatic degradation and remove the adhering ruminal contents. Subsequently the bags were dried at 55 °C for 48 h and reweighed to complete the calculation. The dried samples were kept in a refrigerated room (4 °C) until needed for chemical analysis.

### Chemical analyses

The residues collected from the nylon bags were transferred into labelled containers and ground through a 1 mm screen (Retsch ZM-1; Brinkmann Instruments, Mississauga, ON) for analysis, with the exception of the starch analysis where samples were ground through a 0.5 mm screen. Samples were analysed for dry matter (DM), ash and crude protein (CP) content according to the AOAC [23]. Starch content was analysed using the Megazyme Total Starch Assay Kit (Megazyme International Ltd., Bray, Wicklow, Ireland) based on thermostable α-amylase/amyloglucosidase [24].

### Intestinal protein digestibility

Intestinal protein digestibility (IVCPD) was determined according to the modified *in vitro* method of Calsamiglia and Stern [25–27]. Briefly, dried ground rumen residues containing 15 mg of N after 16 h of ruminal incubation were exposed for 1 h to 10 mL of 0.1 N HCl solution containing 1 g of pepsin/L. The pH was then neutralized with 0.5 mL of 0.5 mol NaOH/L and 13.5 mL of pH 7.8 phosphate buffer containing 37.5 mg of pancreatin, which were added to the solution and incubated at 38 °C for 24 h. After 24 h of incubation, 3 mL of a 100 % (w/v) trichloroacetic acid solution was added to precipitate undigested proteins. The samples were centrifuged, and the supernatant was analysed for N (Kjeldahl method, AOAC 984.13). Intestinal digestion of protein was calculated as TCA-soluble N divided by the amount of N in the 16 h residue sample.

### Statistical analysis

Statistical analyses of the chemical, nutrition and spectral data were performed using the MIXED procedure of SAS (version 9.3). The model used for the chemical and spectral (amide I, II, beta-sheet, alpha-helix, and their ratios etc.), studies was: Y_ijk_ = μ + T_i_ + Y_j_ + e_ijk_. The model used for the rumen and intestinal digestion studies was: Y_ijk_ = μ + T_i_ + Y_j_ + R_j_ + e_ijk_, where, Y_ij_ was an observation of the dependent variable ij; μ was the population mean for the variable; T_i_ was the fixed effect of heat treatment (raw, dry roasting*,* microwave irradiation), Y_j_ and R_i_ were the random year and cows effects, respectively, and e_ij_ and e_ijk_ were the random errors associated with the observation ijk. Tukey method was used for mean separations. Significant level was declared at *P* < 0.05

## Results

### Chemical composition and nutrient availability

Chemical profiles and nutrient availability of raw and heat processed oat seeds (DO and MIO) are presented in Table 1. Significant differences among the treatments for nutrients content were observed except crude protein (*P* > 0.05). Compared with the control (raw), dry roasting did not significantly increased the DM content, while its value was lower than that for the MIO treatment (*P* < 0.05). The MIO resulted in a higher starch content compared to the raw (*P* < 0.05). The in situ nutrient rumen degradation of oat seeds affected significantly by microwave irradiation. The feed rumen residue after 16 h of incubation was higher for the MIO treatment compared to the DO or the raw. The protein intestinal digestibility (Table 1) tended to be affected by heat treatment, while the value obtained for the MIO treatment was higher (*P* < 0.05) and tended to be higher (*P* <0.10) than that of the Raw and DO, respectively

**Table 1:** Chemical profiles and nutrient availability: comparison among raw (RO), dry roasted (DO) and microwave irradiation (MIO) oat seed

Item	Raw (RO)	Oat Heat Treatment		SEM	*P* value	Contrast		
		Dry roasting (DO)	Microwave irradiation (MIO)			Do *vs* MIO *P* value	MIO *vs* RO *P* value	DO *vs* RO *P* value
Chemical Profile, % DM								
DM	90.51 ^b^	92.37 ^b^	96.01 ^a^	0.54	0.012	0.018	0.006	0.094
ASH	3.55 ^a^	2.95 ^b^	3.68 ^a^	0.07	0.012	0.006	0.285	0.011
CP	12.94	12.60	12.25	0.18	0.160	0.266	0.076	0.283
Starch	50.88 ^b^	56.09 ^a^	54.56 ^ab^	0.76	0.036	0.251	0.042	0.017
OM	96.79 ^b^	97.28 ^a^	96.47 ^b^	0.07	0.011	0.005	0.055	0.019
Residue of feed nutrients after 16 h rumen incubation, %								
DM	35.05^b^	28.58^b^	57.20^a^	2.17	0.005	0.003	0.006	0.125
CP	24.49^b^	20.19^b^	69.19^a^	3.08	0.003	0.015	0.002	0.040
Starch	17.21^b^	10.46^b^	43.03^a^	3.98	0.019	0.010	0.018	0.308
OM	33.05^b^	27.89^c^	53.25^a^	0.30	<.0001	<.0001	<.000	0.001
*In vitro* crude protein digestibility in the small intestine, % CP (in ruminally undegraded residues)								
IVCPD	50.06	57.57	72.14	3.94	0.06	0.079	0.029	0.271

*SEM* standard error of mean, *DM* dry matter, *CP* crude protein, *OM* organic matter

^a, b, c^Means with different letters within the same row differ (*P* < 0.05)

### Heat-induced changes in molecular structure characteristics

Results from the molecular structure analyses that are related to protein and starch is shown in Table 2. In the present study, the microwave irradiation decreased (*P* < 0.05) the ratio of α-helix to β-sheet, the amide I and II height, the α-helix, β-sheet, and the amide I to starch area ratios compared with the raw (control). It means that the microwave irradiation increased the ratio of β-sheet to α-helix.

**Table 2:** Molecular structure spectral characteristics using ATR-FT/IR molecular spectroscopy: comparison among raw (RO), dry roasted (DO) and microwave irradiation (MIO) oat seed

Item	Oat Heat Treatment					Contrast		
	Raw (RO)	Dry roasting (DO)	Microwave irradiation (MIO)	SEM	*P* value	Do *vs* MIO *P* value	MIO *vs* RO *P* value	DO *vs* RO *P* value
Protein amide molecular structure spectral profiles (Unit: Absorbance)								
Amide I area	2.82	2.68	1.94	0.30	0.13	0.11	0.06	0.06
Amide II area	0.74	0.71	0.56	0.07	0.15	0.07	0.14	0.13
Ratio amide I to amide II area	3.94^a^	3.61^ab^	3.44^b^	0.11	0.03	0.29	<0.01	<0.01
Amide I height	0.05	0.05	0.03	0.01	0.06	0.05	0.03	0.03
Amide II height	0.02^a^	0.02^ab^	0.01^b^	<0.01	0.03	0.02	0.01	0.01
Ratio amide I to amide II height	2.47	2.45	2.55	0.05	0.36	0.18	0.28	0.28
Protein secondary structure spectral profile								
a-helix (height)	0.05	0.04	0.03	0.01	0.06	0.11	0.02	0.02
β-sheet (height)	0.04^a^	0.03^ab^	0.02^b^	0.004	0.03	0.12	<0.01	<0.01
Ratio a-helix to β-sheet	1.26^b^	1.39^ab^	1.40^a^	0.01	<.0001	0.57	<.0001	<.0001
Starch related molecular structure spectral profiles								
Starch area	7.50	7.92	6.86	0.78	0.643	0.35	0.71	0.57
Starch height	0.18	0.18	0.17	0.02	0.50	0.28	0.88	0.35
Ratio amide I to starch area	0.37^a^	0.34^b^	0.28^c^	0.02	<0.01	0.02	<0.01	<0.01

*SEM* standard error of mean

^a, b, c^Means with different letters within the same row differ (*P* < 0.05)

### Correlations between protein and starch structure characteristics and nutrient profiles

Correlations between protein and starch molecular structure characteristics and nutrient profiles of oat seeds are presented in Table 3. Crude protein found to be positive correlated (R = −0.99, *P* <0.05) with the β-sheet while dry matter was negatively correlated with the ratio amide I to starch area (R = −0.99, *P* < 0.05). The IVCPD was also negatively correlated with α-helix (R = −0.99, *P* <0.01) and ratio of amide I to starch area (R = −0.99, *P* <0.05).

**Table 3:** Correlation between molecular structures (amide I, amide II, their ratio and amide I and starch ratio) and nutrient profiles of processed oat seed

Items	Chemical and nutrient digestion ^a^											
	DM		ASH		CP		OM		Starch		IVCPD	
	R	*P* value	R	*P* value	R	*P* value	R	*P* value	R	*P* value	R	*P* value
Protein molecular profiles												
Amide I height	−0.97	0.150	−0.56	0.624	0.92	0.254	0.74	0.473	−0.32	0.786	−0.97	0.152
Amide I area	−0.98	0.118	−0.51	0.657	0.94	0.221	0.70	0.506	−0.38	0.754	−0.98	0.120
Amide II height	−0.98	0.142	−0.55	0.632	0.93	0.245	0.73	0.482	−0.34	0.770	−0.97	0.144
Amide II area	−0.87	0.334	−0.77	0.441	0.77	0.437	0.90	0.289	−0.05	0.970	−0.86	0.336
α-helix	−0.99	0.003	−0.35	0.772	0.99	0.106	0.56	0.621	−0.54	0.639	−0.99	0.005
β-sheet	−0.99	0.097	−0.20	0.872	0.99	0.007	0.43	0.720	−0.66	0.534	−0.99	0.095
Ratio α-helix to β-sheet	0.80	0.404	−0.28	0.821	−0.89	0.300	0.043	0.973	0.93	0.232	0.81	0.402
Ratio amide I to amide II height	0.86	0.340	0.78	0.435	−0.77	0.444	−0.90	0.284	0.038	0.980	0.86	0.342
Ratio amide I to amide II area	−0.94	0.229	0.006	0.996	0.98	0.126	0.23	0.853	−0.80	0.417	−0.94	0.227
Starch Molecular profiles												
Starch height	−0.88	0.302	−0.74	0.470	0.79	0.413	0.88	0.322	−0.09	0.941	−0.89	0.311
Starch area	−0.73	0.483	−0.89	0.292	0.61	0.580	0.97	0.152	0.18	0.894	−0.73	0.472
Ratio amide I to starch (area)	−0.99	0.034	−0.30	0.812	0.99	0.071	0.52	0.650	−0.58	0.610	−0.99	0.030

^a^ *DM* dry matter, *CP* crude protein, *OM* organic matter, *IVCPD* In vitro crude protein digestibility in small intestine, *R* correlation coefficient

## Discussion

In the present study, compared with the control, dry roasting significantly increased the DM content, indicating that dry treatment decreased the moisture holding capacity of oat seeds. Moreover, the crude protein content did not affected by any heat treatment in accordance with earlier findings [6–10]. Concerning starch, although it plays an important role in the processing of oat based feeds and is an important component of the its grain, the effect of heat treatments on oat starch structure properties is poorly characterized in current literature. The total starch content in our oat samples was affected by each of the heat treatment used, and was in the range that is typically found in oats [28].

The results based the chemical profile and nutrient degradability changes, indicated that the susceptibility of oat seeds to heating methods was distinct. Microwave irradiation was more efficient in altering the protein profile and influences the nutrient ruminal degradability (i.e., nutrient residue) than the dry roasting. The increase of the feed residue after rumen incubation and at the same time the increase in intestinal digestibility of rumen undegradable protein with microwave irradiation can be related to the shift in protein subfractions [7].

No differences found between the raw and the dry roasted seeds on the IVCPD in accordance with Samadi and Yu [7] on soybeans. One possible reason for this is that the temperature or the time for the treatments was not sufficient in order to damage the oat seed protein, and so let any differences estimation using a protocol by Calsamiglia and Stern [25], to be detected. As stated previously by Stern et al. [29], heating above the optimal temperature may overprotect the protein, so that the protein is neither fermented in the rumen nor digested in the small intestine.

Our research study explored the molecular structure related to protein and starch and the effect of heat treatments on their profile. Heat processing reduces protein degradation in the rumen and optimizes protein utilization in ruminants by changing protein molecular structures, such as denaturation, unfolding or uncoiling of a coiled or pleated structure, or the separation of the protein into its subunits, which may then unfold or uncoil [1, 11, 17]. The heat-induced changes in inherent protein structures, (i.e., the ratios of amide I to amide II area, α-helix to β-sheet) affect the rumen and intestinal digestibility of protein by changing protein solubility [6], and the access of microbes and proteolytic enzymes to protein molecules in the gastrointestinal tract of dairy cattle [11, 18]. Even if feeds contain similar protein contents, their nutritive value may be different if the α-helix-to-β-sheet ratios of their protein secondary structures are different [7]. In our study, α-helx and the ratio of α-helix to β-sheet were affected by the heat process and this alteration in the protein structure ratio was probably caused by denaturation of α-helices and β-sheets during the heat treatment.

Moreover compared to control seeds (raw) dry roasting did not affect the protein secondary structure profile in contrast with the results found by Yu [16] on flaxseed. They concluded that dry heating decreased the percentage of α-helix (from 47.1 to 36.1 %), increased the percentage of β-sheet (from 37.2 to 49.8 %), and decreased the α-helix-to-β-ratio. Similar results with our study mentioned by Peng et al. [8] on camelina seeds where, dry heating did not change any of their measured spectral characteristics associated with protein structure compared with raw seeds.

On the other hand, thermal properties of starch play an important role in feed processing and are highly dependent on the starch structure, specifically, the amylose and amylopectin ratio [30]. So, since the physicochemical and digestibility properties of starch are influenced by the starch structure, it would be interesting to know how the heat treatments affect these parameters in oat starch. So, we found that only the ratio amide I to starch area affected by heat processing.

## Conclusion

The results reveal that oat seed is more sensitive to microwave irradiation than dry heating in terms of protein and starch molecular profile and nutrient availability in ruminants.
